# The five times sit-to-stand test predicts achievable exercise intensity during stress echocardiography

**DOI:** 10.1093/ehjimp/qyaf030

**Published:** 2025-03-13

**Authors:** Yasuhide Mochizuki, Yui Kuroki, Mina Shibakai, Ayaka Oda, Sakiko Gohbara, Yumi Yamamoto, Saaya Ichikawa-Ogura, Rumi Hachiya, Eiji Toyosaki, Hiroto Fukuoka, Toshiro Shinke

**Affiliations:** Division of Cardiology, Department of Medicine, Showa University School of Medicine, 1-5-8 Hatanodai, Shinagawa-ku, Tokyo 142-8555, Japan; Ultrasound Examination Centre, Showa University Hospital, Tokyo, Japan; Ultrasound Examination Centre, Showa University Hospital, Tokyo, Japan; Division of Cardiology, Department of Medicine, Showa University School of Medicine, 1-5-8 Hatanodai, Shinagawa-ku, Tokyo 142-8555, Japan; Division of Cardiology, Department of Medicine, Showa University School of Medicine, 1-5-8 Hatanodai, Shinagawa-ku, Tokyo 142-8555, Japan; Division of Cardiology, Department of Medicine, Showa University School of Medicine, 1-5-8 Hatanodai, Shinagawa-ku, Tokyo 142-8555, Japan; Division of Cardiology, Department of Medicine, Showa University School of Medicine, 1-5-8 Hatanodai, Shinagawa-ku, Tokyo 142-8555, Japan; Division of Cardiology, Department of Medicine, Showa University School of Medicine, 1-5-8 Hatanodai, Shinagawa-ku, Tokyo 142-8555, Japan; Division of Cardiology, Department of Medicine, Showa University School of Medicine, 1-5-8 Hatanodai, Shinagawa-ku, Tokyo 142-8555, Japan; Division of Cardiology, Department of Medicine, Showa University School of Medicine, 1-5-8 Hatanodai, Shinagawa-ku, Tokyo 142-8555, Japan; Division of Cardiology, Department of Medicine, Showa University School of Medicine, 1-5-8 Hatanodai, Shinagawa-ku, Tokyo 142-8555, Japan

**Keywords:** exercise stress echocardiography, sarcopenia, five times sit-to-stand test, body composition analyser

## Abstract

**Aims:**

Exercise stress echocardiography (ESE) is becoming increasingly important in assessing heart failure and valvular diseases; however, determining optimal exercise intensity remains challenging, particularly in patients with physical disorders.

**Methods and results:**

A total of 94 patients scheduled for ESE were enrolled in the study. Physical capability was assessed using the five times sit-to-stand test (5-STS), Clinical Frailty Scale, acronyms of the five components, namely strength, assistance with walking, rising from a chair, climbing stairs, and falls (SARC-F) questionnaire, grip strength test, and bioelectrical impedance analysis. In the derivation cohort (*n* = 43), we determined the 5-STS cut-off value to achieving a 25 W load. The effectiveness of this cut-off value was prospectively evaluated in a validation cohort (*n* = 51). In the derivation cohort, the 5-STS predicted achieving a 25 W load using a cut-off of 11.7 s with 91% sensitivity and 70% specificity. In the validation cohort, using 12.0 s as the cut-off demonstrated 98% sensitivity and 88% specificity. The multivariate analysis identified age, sex (female), brain natriuretic peptide, SARC-F, and 5-STS as independent predictors of maximum achieved load. In a multivariate model including bioelectrical impedance parameters, lower limb muscle mass independently influenced maximum achievable load, regardless of age. Patients with optimized 5-STS-based load selection achieved significantly higher peak heart rates and maximum loads than those without.

**Conclusion:**

Sarcopenia-related indices, particularly the 5-STS, effectively and simply predicted achievable exercise intensity during ESE, independent of age and sex. The use of these indices to determine the initial load may help optimize ESE protocols for individual patients.

## Graphical Abstract

**Graphical Abstract:**
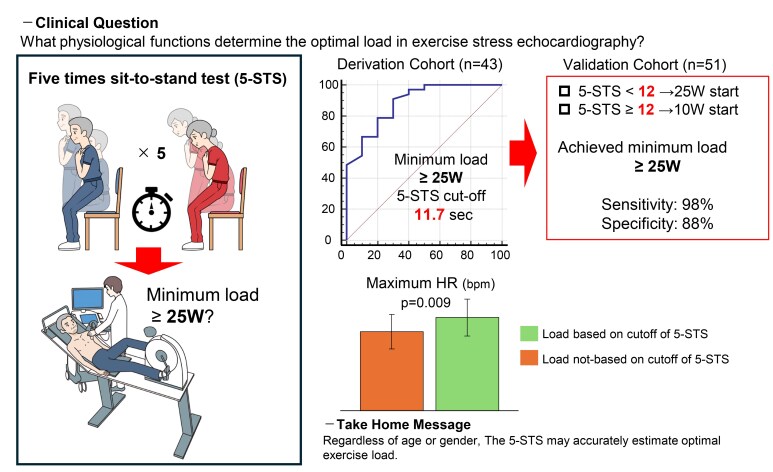

## Introduction

Heart failure (HF) has become a global pandemic and is accelerating in prevalence as the population ages.^1–3^ Epidemiological data suggest that HF is often accompanied by unintentional weight loss, muscle weakness, and decreased physical performance that can influence its course and its prognosis.^4–6^ In such patients, basic physical activities are impaired, making it difficult for themselves and the physicians to objectively assess the symptoms of cardiovascular disease and the differences between rest and exertion.

With the increasing number of patients with HF and valvular disease, the importance of exercise stress echocardiography (ESE) is growing because it helps assess cardiac function under physical stress, revealing abnormalities that may not be apparent at rest, which aids in the diagnosis and management of cardiovascular conditions.^7,8^ In multistage exercise testing using an ergometer, it is common to select a load increase at either 10 W or 25 W increments^9,10^; however, in clinical practice, setting an optimal exercise intensity based solely on age and body shape in patients with HF and sarcopenia is difficult.^11–13^ In ESE, both for valvular disease and ischaemic heart disease, it is generally stated that achieving a workload exceeding 85% of the age-predicted target heart rate (HR) ensures sufficient diagnostic accuracy.^14–16^ Furthermore, the importance of applying an individually tailored workload is emphasized. However, there remains no clear consensus on the specific criteria by which the appropriate exercise intensity should be determined for each patient. If physical capabilities can be easily and objectively evaluated in advance, patients can perform optimal exercises tailored to their activity levels, thereby enhancing ESE with high clinical implications, including improved diagnostic accuracy and more appropriate therapeutic interventions.

Several indicators are available to estimate the intensity of daily activities for patients: scaling to measure the degree of frailty obtained from questionnaires; body composition indicators, such as muscle mass, body fat percentage, visceral fat, and body water content; and the five times sit-to-stand test (5-STS), which is a simple test to measure agility.^17–19^ Therefore, we hypothesized that if sarcopenia-related indices measured prior to ESE demonstrate significant correlations with exercise performance, then these measurements could serve as reliable predictors of maximum exercise intensity during ESE.

## Methods

### Study population

**Figure 1:**
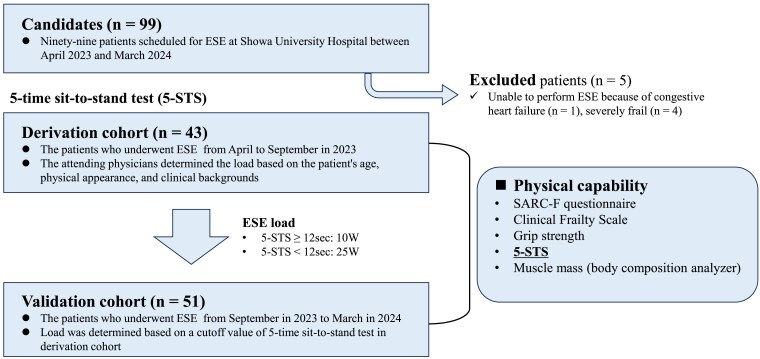
Patient enrolment. Of the 99 patients scheduled for exercise stress echocardiography at the Showa University Hospital from April 2023 to March 2024, 94 were eligible for analysis and were divided into two cohorts: derivation (*n* = 43, age/visual assessment-based load) and validation (*n* = 51, five times sit-to-stand test-based load) cohorts.

A total of 99 patients scheduled for ESE at the Showa University Hospital between April 2023 and March 2024 were initially included in the study. Five patients were unable to undergo ESE because of congestive HF (*n* = 1) and severe frailty (*n* = 4). In this study, we investigated the significance of determining the exercise intensity based on the results of the 5-STS, as outlined in the ESE method. There are no prior studies; however, based on our previous experience with ESE, the positive (≥25 W) to negative (<25 W) ratio was estimated at 3:1. With an expected area under curve (AUC) of 0.80 and null hypothesis value of 0.5, the minimum total sample size required was calculated as 34 subjects across both groups (α=0.05, β=0.2). Accordingly, the derivation cohort sample size was set to exceed 40 patients. As shown in *Figure 1*, the first 43 patients were assigned to a derivation cohort, wherein the exercise load for ESE was determined based on age and visual assessment alone, without consideration of the 5-STS results. In the validation cohort, required total sample size was calculated as 34 with a proportion of 90% and a null hypothesis value of 0.7 (α=0.05, β=0.2). Accordingly, a minimum of 40 patients was targeted. Subsequently, we enrolled 51 patients in the validation cohort, wherein the exercise load was specifically allocated according to their 5-STS performance metrics. The final analysis included 94 patients who successfully underwent ESE. The baseline characteristics, including the demographics and laboratory data, were retrospectively collected from the medical records. This study was approved by the local ethics committee of Showa University Research Administration Center (Approval No. 2024-201-A) and was conducted in accordance with the Declaration of Helsinki. Informed consent was obtained in the form of an opt-out on the website, and those who refused were excluded.

### Assessment of physical capability and body composition

**Figure 2:**
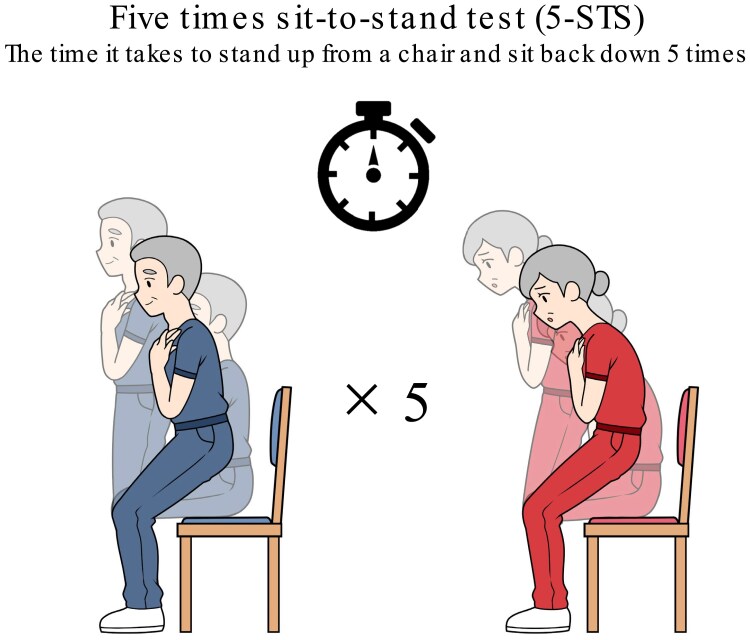
The five times sit-to-stand test (5-STS) measures the time required to stand up from a seated position and sit back down five times consecutively as quickly as possible without using the hands. A result of 12 s or longer (5-STS ≥ 12 s) suggests the possibility of sarcopenia.

Each patient was assessed using the Clinical Frailty Scale (CFS)^20^ and acronyms of the five components, namely strength, assistance with walking, rising from a chair, climbing stairs, and falls (SARC-F) screening questionnaire for sarcopenia.^17^ Grip strength was measured as an indicator of muscle strength, and body composition, including muscle, fat, bone mineral, and water was evaluated using a commercially available bioelectrical impedance analyser (InBody770, InBody Co., Ltd., Seoul, Korea). Physical agility was assessed using the 5-STS,^19^ which measures the time (in seconds) required for a participant to stand up and sit back down in a chair five consecutive times (*Figure 2*).

### ESE

The ESE was performed using a supine ergometer. Following established guidelines, the protocol began with an initial load of either 10 W or 25 W.^9,10^ The exercise intensity was increased using the Bruce protocol with load increments every 3 min, and the ESE was continued until the symptom-limited maximum exertion was achieved. As shown in *Figure 1*, to determine the cut-off value of the 5-STS for identifying whether 25 W of exercise load was achievable, we conducted a retrospective analysis using patients (*n* = 43) who underwent ESE by 7 September 2023 as the derivation cohort. During this cohort period, the initial exercise load intensity (either 10 W or 25 W) was determined by the examining physician, who was blinded to the results of the 5-STS, based on the age, physical appearance, and clinical background of the patients. Then, we defined patients who underwent ESE after 8 September 2023 (*n* = 51) as the validation cohort and assessed the validity of the 5-STS for determining the initial exercise load intensity. Based on the cut-off value from the 5-STS, we determined whether to use 10 W or 25 W increments for the load on the ESE. The main outcome was whether the exercise load setting for ESE based on the cut-off value of 5-STS was useful, while maximum exercise load and maximum HR were assessed as secondary outcomes representing the quality of the final exercise load.

### Statistics analysis

Continuous variables were expressed as mean with standard deviation for normally distributed data and medians with interquartile ranges for non-normally distributed data. Categorical variables were expressed as frequencies and percentages. The parameters of the two subgroups (derivation and validation cohorts) were compared using Student’s *t*-test or the Mann–Whitney *U* test, as appropriate. Proportional differences were evaluated using Fisher’s exact test or the chi-squared test. The relationships between two variables were analysed using linear regression and expressed as Pearson’s correlation coefficients. The cut-off value for the 5-STS was determined using receiver operating characteristic (ROC) curve analysis. Multivariate regression analysis of factors related to the maximum achieved load intensity was performed using stepwise selection of variables that showed a significant correlation in the univariate analysis. Statistical significance was defined as *P* < 0.05. MedCalc Version 23.0.6 (MedCalc Software Ltd, Mariakerke, Belgium) was used for all analyses.

## Results

### Patient backgrounds

*Table 1* summarizes the patient characteristics, including demographic data, ESE parameters, and body composition measurements across the three groups: total study population (*n* = 94), derivation cohort (*n* = 43), and validation cohort (*n* = 51). Statistical comparisons were performed between the derivation and validation cohorts to assess the between-group differences. In the total study population, there were 48 women (51%), the median age was 76.5 (63.0–83.0) years, and 75 (79.8%) patients had mitral valve regurgitation. In the blood examination, the median albumin and brain natriuretic peptide (BNP) levels were 4.0 (3.8–4.2) g/dL and 117.3 (57.7–237.4) pg/mL, respectively. A total of 53 (56.4%) and 50 (53.1%) patients were taking renin-angiotensin-aldosterone system inhibitors and beta-blockers, respectively. The median maximum achieved load according to the ESE was 50 (25–75) W. The reasons for terminating the exercise were leg fatigue and dyspnoea in 73 (77.7%) and 41 (43.6%) patients, respectively. The median Borg scale score was 14 (13–17) and 15 (13–17) for respiration and leg fatigue, respectively. Thirteen (13.8%) patients had a pre-specified target HR. In the comparison between the two cohorts, the only significant difference was the higher prevalence of mitral regurgitation as an indication for ESE in the derivation cohort than in validation cohort (90.7% vs. 70.6%, *P* = 0.016). There were no significant differences in the patient characteristics, physical functional parameters, or body composition measurements between the two cohorts, indicating comparable study populations.

**Table 1:** Patient characteristics in the derivation and validation cohort

	Entire patient (*n* = 94)	Derivation (*n* = 43)	Validation (*n* = 51)	*P*-value*
Clinical data				
Age, years	76.5 (63.0–83.0)	77.0 (68.0–83.0)	75.0 (62.5–82.0)	0.514
Sex (female), *n* (%)	48 (51.0)	19 (44.0)	29 (56.8)	0.223
Height, cm	157.4 ± 9.3	158.1 ± 9.1	158.9 ± 9.5	0.702
Weight, kg	56.7 ± 11.7	56.5 ± 10.5	57.9 ± 12.7	0.554
Body mass index, kg/m^2^	22.5 ± 3.5	22.5 ± 3.5	22.8 ± 3.5	0.743
Pacemaker, *n* (%)	7 (7.4)	5 (11.6)	2 (3.9)	0.161
Blood exam				
Hb, g/dL	12.6 ± 1.8	12.4 ± 1.7	12.6 ± 2.0	0.653
Albumin, g/dL	4.0 (3.8–4.2)	4.0 (3.8–4.2)	4.1 (3.7–4.3)	0.618
BNP, pg/mL	117.3 (57.7–237.4)	130.6 (65.4–241.2)	88.0 (55.1–224.8)	0.364
Medications				
ARNI/ARB/ACE-I, *n* (%)	53 (56.4)	27 (62.8)	26 (51.0)	0.253
β-blocker, *n* (%)	50 (53.1)	26 (61.9)	24 (47.1)	0.155
Loop diuretics, *n* (%)	37 (39.4)	20 (46.5)	17 (33.3)	0.195
SGLT2-I, *n* (%)	35 (37.2)	20 (46.5)	15 (29.4)	0.089
MRA, *n* (%)	32 (34.0)	18 (41.9)	14 (27.5)	0.144
CCB, *n* (%)	24 (25.5)	11 (25.6)	13 (25.5)	0.992
Measurements in ESE				
Peak achieved load, Watt	50 (25–75)	50 (30–75)	50 (25–75)	0.712
Maximum HR, bpm	107.0 ± 22.5	105.0 ± 21.4	111.1 ± 23.2	0.187
Maximum BP, mmHg	165.5 ± 29.9	165.2 ± 29.5	168.2 ± 30.4	0.629
Borg scale (respiration)	14.0 (13.0–17.0)	15.0 (13.0–19.0)	13.0 (13.0–15.5)	0.073
Borg scale (leg)	15.0 (13.0–17.0)	15.0 (13.0–17.0)	15.0 (13.0–17.0)	0.657
Indication for ESE				
MR, *n* (%)	75 (79.8)	39 (90.7)	36 (70.6)	0.016
MS, *n* (%)	6 (6.4)	2 (4.7)	4 (7.8)	0.530
AR, *n* (%)	2 (2.1)	1 (2.3)	1 (2.0)	0.276
AS, *n* (%)	1 (1.1)	0	1 (2.0)	0.359
PR, *n* (%)	1 (1.1)	1 (2.3)	0	0.276
PH, *n* (%)	4 (4.3)	0	4 (7.9)	0.062
TR, *n* (%)	2 (2.1)	0	2 (3.9)	0.192
HFpEF, *n* (%)	2 (2.1)	0	2 (3.9)	0.192
HCM, *n* (%)	1 (1.1)	0	1 (2.0)	0.359
Indications for termination				
Lower limb fatigue, *n* (%)	73 (77.7)	31 (72.1)	42 (82.4)	0.237
Dyspnoea, *n* (%)	41 (43.6)	23 (53.5)	18 (35.3)	0.078
Achievement of THR, *n* (%)	13 (13.8)	5 (11.6)	8 (15.7)	0.575
Others, *n* (%)	3 (3.2)	1 (2.3)	2 (3.9)	0.663
Physical function				
5-STS, s	9.5 (7.6–11.0)	9.5 (8.2–11.5)	9.5 (7.5–10.7)	0.354
5-STS ≥12 s, *n* (%)	18 (19.0)	10 (23.2)	8 (15.7)	0.433
SARC-F, points	1 (0.0–2.0)	1 (0.0–2.5)	1 (0.0–2.0)	0.390
Clinical Frailty Scale	3 (2.0–3.0)	3 (3.0–3.0)	3 (2.0–4.0)	0.962
Grip strength, kg	22.9 ± 8.1	22.9 ± 8.2	22.7 ± 8.2	0.892
Body composition analyser				
Skeletal muscle mass index	8.6 ± 1.2	8.9 ± 1.4	8.4 ± 1.1	0.116
Lower limb muscle mass, kg	6.0 ± 1.6	6.1 ± 1.7	5.9 ± 1.6	0.753
Protein mass, kg	7.9 ± 1.6	8.1 ± 1.7	7.8 ± 1.6	0.355
Skeletal muscle mass, kg	21.8 ± 5.0	22.4 ± 5.1	21.4 ± 4.9	0.353
Bone mineral content, kg	2.4 ± 0.4	2.5 ± 0.4	2.4 ± 0.4	0.072
Body fat, %	15.8 ± 6.0	15.5 ± 5.3	16.0 ± 6.5	0.719

5-STS, 5-times sit-to-stand test; ACE-I, angiotensin-converting enzyme inhibitor; AR, aortic valve regurgitation; ARB, angiotensin II receptor blocker; ARNI, angiotensin receptor neprilysin inhibitor; AS, aortic valve stenosis; BNP, brain natriuretic peptide; BP, blood pressure; CCB, calcium channel blocker; HCM, hypertrophic cardiomyopathy; HFpEF, heart failure with preserved ejection fraction; HR, heart rate; MR, mitral valve regurgitation; MRA, mineralocorticoid receptor antagonist; MS, mitral valve stenosis; PH, pulmonary hypertension; PR, pulmonary valve regurgitation; SARC-F, Acronyms of five components: Strength, Assistance walking, Rise from a chair, Climb stairs, and Falls; SGLT2-I, sodium-glucose co-transporter 2 inhibitor; THR, target heart rate; TR, tricuspid valve regurgitation.

^*^The *P*-value indicates the statistical significance of differences in patient backgrounds between the derivation and validation cohorts.

### Association between the 5-STS and ESE in the derivation cohort

**Figure 3:**
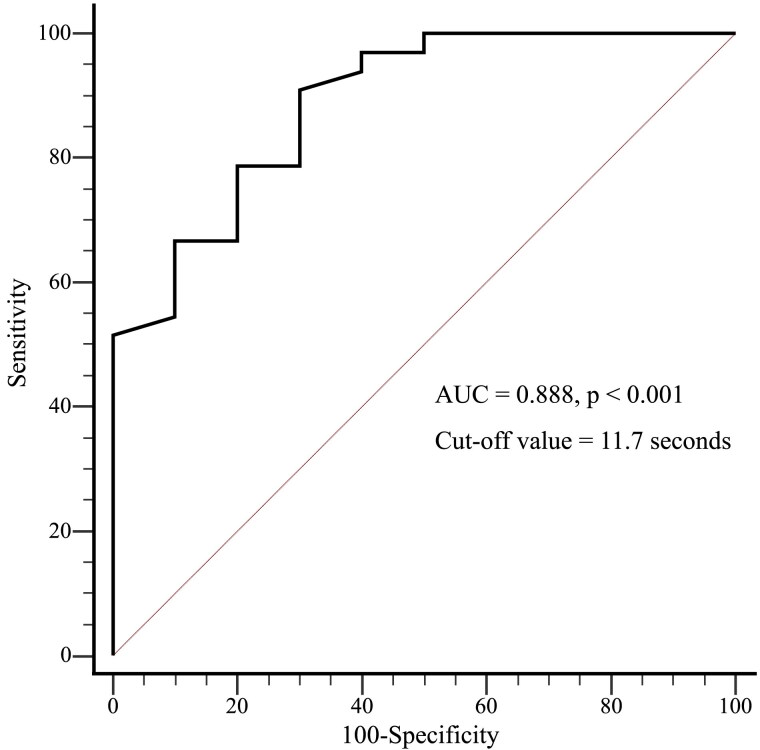
In the ROC analysis of the derivation cohort (*n* = 43), a 5-STS of 11.7 s demonstrated a sensitivity of 91% and specificity of 70% for predicting the ability to achieve a workload of ≥25 W during exercise stress echocardiography.

The 5-STS was safely performed prior to ESE in all patients. In the derivation cohort, the multiple logistic regression analysis that was adjusted for age and sex demonstrated that the 5-STS, SARC-F score, grip strength, BNP level, and muscle mass of the lower limbs were significantly associated with achieving at least a 25 W load (*Table 2*). Among these factors, the 5-STS predicted a load of 25 W or more when the cut-off value was set at 11.7 s with a sensitivity of 91% and specificity of 70% (*Figure 3*, AUC = 0.888, *P* < 0.001), which is close to the 12.0-second cut-off value for diagnosing sarcopenia.^21^

**Table 2:** Logistic regression for the achievement of at least 25 W load in the derivation cohort

Covariate	Multivariate analysis^a^		
	OR	95% CI	*P-*value
SARC-F, points	0.569	0.333–0.974	0.040
Grip strength, kg	2.203	1.238–3.922	0.007
CFS	0.531	0.211–1.334	0.178
5-STS, sec	0.456	0.254–0.818	0.008
BNP, pg/mL	0.992	0.986–0.999	0.026
Bone mineral content, kg	2.461	0.135–44.78	0.543
Body fat, kg	1.007	0.905–1.121	0.899
Skeletal muscle mass, kg	1.719	0.962 -3.071	0.067
Muscle mass of lower limbs, kg	5.135	1.122–23.50	0.035

^a^Adjusted for age and sex.

5-STS, 5-times sit-to-stand test; BNP, brain natriuretic peptide; CFS, clinical frailty scale; CI, confidence interval; OR, odds ratio; SARC-F, Acronyms of five components: Strength, Assistance walking, Rise from a chair, Climb stairs, and Falls.

In the validation cohort (*n* = 51), the ESE was performed with load increments of 25 W and 10 W for patients with 5-STS <12 and ≥12 s, respectively, to validate the appropriateness of this cut-off value. In this prospective validation (*n* = 51), among the eight patients (16%) with 5-STS ≥12 s, only one patient achieved an exercise load of ≥25 W. Among the 43 patients with 5-STS <12 s, only one patient failed to achieve a 25 W load; thus, the pre-specified cut-off value of 5-STS ≥12 predicted the inability to achieve a load of ≥25 W with a sensitivity of 98% and specificity of 88% (AUC = 0.926, *P* < 0.001).

### Variables that correlated with the maximum achieved load intensity

**Figure 4:**
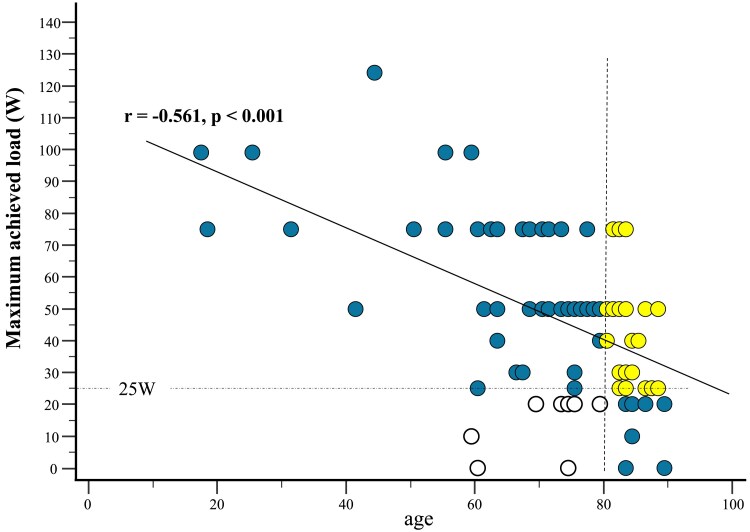
The relationship between age and maximum achieved load. A significant negative correlation was observed between the age and maximum load (*r* = −0.561, *P* < 0.001). Among patients aged over 80 years, 20 out of 37 individuals achieved a load of ≥25 W. Conversely, eight patients under the age of 80 were unable to achieve a load of 25 W.

The correlations between patient parameters and the maximum achieved load for the pooled cohort (derivation and validation groups combined) are shown in *Table 3*. The maximum achieved load was negatively correlated with the BNP (*r* = −0.424, *P* < 0.001), 5-STS (*r* = −0.573, *P* < 0.001), CFS (*r* = −0.496, *P* < 0.001), and SARC-F (*r* = −0.600, *P* < 0.001) scores, while a positive correlation was observed with grip strength (*r* = 0.672, *P* < 0.001). Although the maximum achieved load showed an inverse correlation with age (*r* = −0.561, *P* < 0.001), this association was not strong (*Figure 4*). Among elderly patients aged ≥80 years, 20 out of 37 patients were able to achieve an exercise load of ≥25 W, while eight patients aged <80 years failed to achieve a 25 W load. No statistically significant age threshold was found in the ROC analysis when evaluating the feasibility of achieving a 25 W load (AUC = 0.674, *P* = 0.108).

**Table 3:** The association of patient backgrounds with maximum achieved workload (watt) in ESE

All patients (n = 94)	Correlation coefficient
Age	*r* = −0.561（*P* < 0.001）
Sex (female)	*r* = −0.321（*P* = 0.002）
Body weight	*r* = 0.284（*P* = 0.001）
BNP	*r* = −0.424（*P* < 0.001）
5-STS	*r* = −0.573（*P* < 0.001）
SARC-F	*r* = −0.600（*P* < 0.001）
Clinical Frailty Scale	*r* = −0.496（*P* < 0.001）
Grip strength	*r* = 0.672（*P* < 0.001）

5-STS, 5-times sit-to-stand test; BNP, brain natriuretic peptide; ESE, exercise stress echocardiography; SARC-F, Acronyms of five components: Strength, Assistance walking, Rise from a chair, Climb stairs, and Falls.

### Association between body composition and peak load in the ESE

Seven patients (7%) were unsuitable for measurements with a bioelectrical impedance analyser due to having implanted devices, such as pacemakers or implantable cardioverter-defibrillators. Among the parameters measured using the body composition analyser, muscle mass in both lower limbs (*r* = 0.466, *P* < 0.001), skeletal muscle mass (*r* = 0.474, *P* < 0.001), protein mass (*r* = 0.480, *P* < 0.0001), and bone mineral content (*r* = 0.314, *P* = 0.004) were positively correlated with the maximum load achieved in ESE (*Table 4*).

**Table 4:** Body composition in relation to maximum achieved workload (watt) in ESE

All patients (*n* = 94)	Correlation coefficient
Skeletal muscle mass	*r* = 0.474（*P* < 0.001）
Lower limb muscle mass	*r* = 0.466（*P* < 0.001）
Protein mass	*r* = 0.480（*P* < 0.001）
Bone mineral	*r* = 0.314（*P* = 0.004）
Body fat	*r* = 0.040（*P* = 0.733）

### Multivariate analysis of factors determining the maximum achievable load intensity

The results of the univariate and multivariate regression analyses of the factors related to the maximum achieved load intensity are presented in *Table 5*. In the multiple regression analysis model of the patient backgrounds and sarcopenia diagnostic indicators (*Table 5, model A*), the BNP, SARC-F, and 5-STS were significantly associated with the achievable maximum load, independent of age and sex. Another multiple regression model that included the body composition using a bioelectrical impedance analyser (*Table 5, model B*) revealed that the muscle mass of both lower limbs (β=0.39, *P* < 0.001) was an independent factor for determining achievable maximum load even after adjusting for age.

**Table 5:** Univariate and multivariate regression analysis for determinants associated with achieved maximum exercise load

	Univariate		Multivariate	
Variable	β-Coefficient	*P*-value	β-Coefficient	*P*-value
**Model A**, patient background and diagnostic criteria for sarcopenia				
Age, year	−0.56	<0.001	−0.41	<0.001
Sex (female)	−0.32	0.002	−0.24	<0.001
BNP, pg/mL	−0.40	<0.001	−0.18	0.011
Clinical Frailty Scale	0.58	<0.001		
SARC-F, points	−0.59	<0.001	−0.25	0.010
5-STS, s	−0.57	<0.001	−0.31	0.001
Grip strength, kg	−0.67	<0.001		
**Model B**, Body composition measured by bioelectrical impedance analyzer				
Age, year	−0.56	<0.001	−0.53	<0.001
Sex (female)	−0.32	0.002		
Bone mineral content, kg	0.29	0.007		
Skeletal muscle mass, kg	0.47	<0.001		
Muscle mass of lower limbs, kg	0.46	<0.001	0.39	<0.001
Body fat, kg	−0.04	0.732		

β, standardized β; 5-STS, 5-times sit-to-stand test; BNP, brain natriuretic peptide; SARC-F, Acronyms of five components: Strength, Assistance walking, Rise from a chair, Climb stairs, and Falls.

### Optimal load assessment based on an appropriate physical function

In the overall cohort, two groups were defined as follows: the optimal load group, which underwent ESE at 25 W increments when the 5-STS was <12 s and at 10 W increments when the 5-STS was ≥12 s, and the non-optimal load group, which did not meet these criteria. Patients in the optimal load group (*n* = 80) achieved significantly higher peak HR (111 ± 22 bpm vs. 94 ± 21 bpm, *P* = 0.009), double products [17748 (14060–22493) mmHg × bpm vs. 14263 (12763–16611) mmHg × bpm, *P* = 0.03], and maximum load intensity [50.0 (25.0–75.0) W vs. 40.0 (30.0–47.5) W, *P* = 0.030] compared with the non-optimal load group (*n* = 14; *Table 6*); however, there were no significant differences in the Borg scores for dyspnoea or leg fatigue between the groups.

**Table 6:** Differences in physiological responses to exercise load between two groups based on the 5-STS

	Non-optimal load (*n* = 14)	Optimal load group (*n* = 80)	*P-*value
Peak achieved load, Watt	40.0 (30.0–47.5)	50.0 (25.0–75.0)	0.030
Borg scale for dyspnoea, points	15.0 (13.0–17.0)	14.0 (13.0–16.3)	0.625
Borg scale for leg fatigue, points	15.0 (13.0–17.0)	15.0 (13.0–17.0)	0.995
Maximum systolic BP, mmHg	160.6 ± 23.5	167.9 ± 30.9	0.405
Maximum HR, bpm	93.9 ± 20.6	110.8 ± 22.0	0.009
Maximum DP, mmHg·bpm	14262.5 (12762.8–16610.8)	17748.0 (14060.3–22493.3)	0.030
Maximum HR/target HR, %	77.3 ± 18.5	87.6 ± 16.4	0.036

BP, blood pressure; DP, double product = maximum systolic blood pressure × heart rate; HR, heart rate.

## Discussion

A multistage protocol that increases the load in 25 W increments on a supine ergometer is currently used as the physiological loading method in ESE; however, for elderly individuals or those with limited lower-extremity muscle strength, a multistage method with 10 W increments or a ramp protocol is recommended.^8–10^ There are no established criteria for muscle strength assessment to guide the current protocol selection. Consequently, with the aging population, there have been an increasing number of cases wherein patients either fail to complete the test due to excessive load or fail to achieve adequate stress due to insufficient exercise intensity.

Patients with 5-STS of <12 s could almost certainly withstand a minimum exercise load of 25 W; hence, the 5-STS is useful for selecting an appropriate exercise load that matches the physical capabilities of the patients, regardless of age. In addition to a 5-STS of ≥12 s, other sarcopenia-related indicators, such as the SARC-F score, provide valuable information prior to conducting ESE, which allow clinicians to set an exercise load that is appropriate for the physical capacity of each patient. Quantitative body metrics, such as muscle mass measured using body composition analysers, are associated with the maximum achievable load in ESE. The muscle mass of both lower legs was found to be an independent factor associated with the load after adjusting for age.

### The role and position of 5-STS in physical functional assessment

Among the previously reported physical assessments, namely the Short Physical Performance Battery, usual gait speed, 6-minute walk test, stair-climb power test, timed-up-and-go test, and 5-STS, the usual gait speed was the most frequently used measure and demonstrated strong associations with the onset of disability, severe mobility limitations, and mortality.^21^ A cut-off time of 11.6 s for the 5-STS has been reported to correspond to a gait speed of 1.0 m/s, making the 5-STS a proposed substitute for gait speed in sarcopenia diagnosis.^22^ Furthermore, the 5-STS was also reported to correlate with the stair-climb power test.^23^ Congruently, the Asian Working Group for Sarcopenia 2019 guidelines recommend a cut-off of ≥12 s for 5-STS to indicate low physical performance.^21^ The 5-STS is a practical assessment tool for measuring physical function that requires only a chair and a stopwatch. This test is relatively easy to perform and demonstrates high reproducibility and reliability. Studies have reported that the 5-STS not only reflects knee extension strength but also encompasses knee flexion strength, ankle muscle strength, psychological elements, such as anxiety and vitality, and sensory components, such as proprioception and tactile intensity.^24–26^ Several studies have shown that the 5-STS is an effective predictor of exercise tolerance in older adults.^27,28^ A study by Adsett *et al*.^29^ showed that the 5-STS is a reliable measure of functional exercise capacity in patients with HF. In the present study, the correlation with the maximum load intensity of the 5-STS was moderate, suggesting that multiple factors may be involved in determining exercise tolerance. Indeed, as demonstrated in the multivariate analysis, even after adjusting for age, lower limb muscle strength and the higher BNP level remained independent determinants of maximum load. From the perspective that the initial load setting for the 5-STS can likely be easily stratified, it is considered a valuable indicator that should be known prior to ESE.

### The correlation between sarcopenia diagnostic factors and exercise load intensity

Sarcopenia is a progressive skeletal muscle disorder characterized by the loss of muscle mass, decline in muscle strength, and reduced physical performance. It is a condition primarily observed in older adults (aged 60 and above); however, its progression can be accelerated by factors such as physical inactivity, poor nutrition, chronic illnesses, and hormonal imbalances.^11,30^ The SARC-F questionnaire is comprised of five questions [S (strength), A (assistance with walking), R (rising from a chair), C (climbing stairs), and F (falls)] that is designed to evaluate the comprehensive risk of sarcopenia.^18^ Each component was scored on a scale of 0–2, with a total score of 4 or higher indicating a high risk of sarcopenia.^21^ The inclusion of the item regarding the ability to rise from a chair (R) suggested a significant correlation between the SARC-F and 5-STS. The multivariate regression analysis identified the SARC-F as a strong determinant of the maximum achieved exercise load, underscoring its relevance as a preliminary assessment tool prior to conducting ESE. In contrast, lower limb muscle mass quantified using bioelectrical impedance analysis was identified as an associated determinant of maximum load intensity in the multivariate analysis, excluding sarcopenia diagnostic indicators independent of age. These findings suggest that actual lower-limb muscle mass, in addition to chronological age, is associated with physical performance in patients with HF. Conversely, a previous study reported that lower-limb muscle mass and strength in elderly individuals do not necessarily correlate with improved exercise capacity.^31^ Our results suggest the necessity for a comprehensive assessment that incorporates muscle mass, strength, and simple diagnostic indicators used for sarcopenia evaluation, such as the 5-STS and SARC-F.

### Potential of a 5-STS-based classification to achieve optimal load in ESE

Patients were divided into the optimal load and non-optimal load groups based on their performance in the 5-STS. The results showed that the optimal load group had a significant increase in the HR relative to the target HR, as well as a greater increase in the double product. In ESE, for the diagnosis of ischaemic heart disease, achieving a target HR of at least 80% of the maximum predicted HR is generally required^10,32^; however, there is limited evidence regarding appropriate HR responses in other conditions, such as valvular heart disease.^15^ On the other hand, there was no difference in the Borg scale between the two groups. It was reported that in elderly people, correlation coefficients between the Borg scale and HR and maximum oxygen uptake are lower than those in young adults.^33^ Indeed, there appears to be a gap between physiological responses and subjective perception in elderly patients. Potential reasons for this include cognitive decline, the influence of arrhythmias and medical treatment for HF. Therefore, objective evaluation using physiological parameters becomes particularly important in HF patients, who are predominantly elderly. An appropriate exercise load is an important factor, and the use of the 5-STS to determine exercise intensity may provide an optimal load.

### Limitations

This was a relatively small-scale investigation conducted at a single centre; thus, the sarcopenia diagnostic index deemed useful for determining the exercise echocardiography load in this investigation requires further validation in a multicentre study. In the validation cohort, the physicians were not blind to the 5-STS values; therefore, there is a possibility that potential bias was introduced regarding the intensity of the load in ESE. Furthermore, most cases in the current study included patients with valvular heart disease primarily involving mitral regurgitation. However, a logistic regression analysis indicated that the prevalence of mitral regurgitation was not associated with achieving a workload of ≥25 W. Validation in a broader population encompassing ischaemic heart disease, HF with preserved and reduced ejection fraction, pulmonary hypertension, and diverse cardiomyopathies is beneficial. The maximum load in ESE is not a pure indicator of physical capacity, as it is intentionally controlled by the examining physician based not only on physical appearance but also on the severity of symptoms, type of valvular disease, and degree of pulmonary hypertension. In this study, no other functional exercise tests were performed prior to ESE. To further substantiate the utility of the 5-STS in determining workload, comparative studies incorporating additional functional assessments are warranted. Further studies addressing these limitations are essential to validate our findings.

## Conclusions

Diagnostic indices including the 5-STS related to sarcopenia can accurately stratify the physical capacity of patients with HF and predict the optimal exercise intensity for ESE. Utilizing these indices may facilitate the achievement of an appropriate load during patient evaluation.

## Data Availability

The datasets generated and analysed during the current study are available from the corresponding author upon reasonable request.

## Lead author biography

Dr Yasuhide Mochizuki, the lead author, began his career in cardiovascular medicine after graduating from Kansai Medical University in 2005. He specialized in cardiovascular imaging, focusing on echocardiography from 2012, and has made notable academic contributions. Since 2019, he has served as Senior Lecturer at Showa University, focusing on research and education in valvular heart disease, cardiomyopathy, and heart failure.
